# Anti-schistosomal immunity to core xylose/fucose in N-glycans

**DOI:** 10.3389/fmolb.2023.1142620

**Published:** 2023-04-04

**Authors:** Nina Salinger Prasanphanich, Kristoffer Leon, W. Evan Secor, Charles B. Shoemaker, Jamie Heimburg-Molinaro, Richard D. Cummings

**Affiliations:** ^1^ Department of Biochemistry, Emory University School of Medicine, Atlanta, GA, United States; ^2^ Division of Parasitic Diseases and Malaria, Centers for Disease Control and Prevention, Atlanta, GA, United States; ^3^ Department of Infectious Disease and Global Health, Tufts University Cummings School of Veterinary Medicine, North Grafton, MA, United States; ^4^ National Center for Functional Glycomics, Department of Surgery, Beth Israel Deaconess Medical Center, Harvard Medical School, Boston, MA, United States

**Keywords:** schistosomiasis, immunity, N-glycans, antigens, core fucose, core xylose

## Abstract

Schistosomiasis is a globally prevalent, debilitating disease that is poorly controlled by chemotherapy and for which no vaccine exists. While partial resistance in people may develop over time with repeated infections and treatments, some animals, including the brown rat (*Rattus norvegicus*), are only semi-permissive and have natural protection. To understand the basis of this protection, we explored the nature of the immune response in the brown rat to infection by *Schistosoma mansoni*. Infection leads to production of IgG to parasite glycoproteins with complex-type N-glycans that contain a non-mammalian-type modification by core α2-Xylose and core α3-Fucose (core Xyl/Fuc). These epitopes are expressed on the surfaces of schistosomula and adult worms. Importantly, IgG to these epitopes can kill schistosomula by a complement-dependent process *in vitro*. Additionally, sera from both infected rhesus monkey and infected brown rat were capable of killing schistosomula in a manner inhibited by glycopeptides containing core Xyl/Fuc. These results demonstrate that protective antibodies to schistosome infections in brown rats and rhesus monkeys include IgG responses to the core Xyl/Fuc epitopes in surface-expressed N-glycans, and raise the potential of novel glyco-based vaccines that might be developed to combat this disease.

## Introduction

Schistosomiasis, caused by infection with parasitic helminths of the genus *Schistosoma*, infects over two hundred million people worldwide and causes up to seventy million disability-adjusted-life years (DALYs)—more DALYs than those caused by malaria (Hotez et al., 2009; Hotez et al., 2010). Mass drug administration programs are critical to reducing the burden of disease, but may be insufficient for global elimination (Lo et al., 2015; Lo et al., 2016; Lo et al., 2017; Lo et al., 2022; Ogongo et al., 2022). *Science* magazine identified schistosomiasis as one of the ten diseases for which a vaccine is most needed (Cohen, 2016). Despite this need, only three candidates are currently in clinical trials, and most candidates have shown modest efficacy in the pre-clinical pipeline (Gonçalves de Assis et al., 2015; Molehin et al., 2016; Siddiqui and Siddiqui, 2017; Zhang et al., 2018; Keitel et al., 2019; Molehin et al., 2022). Novel vaccine targets are therefore urgently needed. Many different mammals can host schistosomes, including rodents, non-human primates, and bovines. Some species, like humans, become chronically infected, while others can clear the worms soon after infection (Warren and Peters, 1967; Cheever, 1969; Wilson et al., 2008; Yang et al., 2012). Resistant species provide a natural example of targets and mechanisms of protective immunity that could be exploited in vaccines for humans.

Rodents display varying levels of susceptibility to *Schistosoma mansoni* infection, even when a similar number of cercariae penetrate the skin (Warren and Peters, 1967). Laboratory rats (*Rattus norvegicus* or brown rats) are considered a semi-permissive host, as they are highly resistant to a large dose (500 cercariae) and relatively tolerant to lower doses (10–50 cercariae) (Phillips et al., 1975). At any dose, lab rats dramatically reduce the number of worms, typically to 0%–10%, by 8 weeks post infection. Egg-laying is minimal, and eggs are infertile (Warren and Peters, 1967; Knopf et al., 1977; Jourdane and Imbert-Establet, 1980; Imbert-Establet, 1982; Alarcón de Noya et al., 1997). Rats do not manifest signs of infection and will clear a second infection more rapidly than the first (Warren and Peters, 1967; Phillips et al., 1975; Knopf et al., 1977; Phillips et al., 1977; Cioli et al., 1978; Jourdane and Imbert-Establet, 1980; Imbert-Establet, 1982; Alarcón de Noya et al., 1997; Sepulveda et al., 2010). By contrast, between 8–20 weeks post infection, mice harbor roughly 50% of the worms to which they were exposed, and exhibit hepatosplenomegaly due to thousands of deposited eggs that move into tissues (Warren and Peters, 1967; Cheever, 1969). Mice develop partial immunity after vaccination with irradiated cercariae, but still become chronically infected upon exposure to live cercariae (Bickle, 2009).

Both immunological and physiological factors likely play roles in the differential responses of rodents to *S. mansoni* during the early weeks of infection. Targeting of migrating schistosomula within the first few days is critical for protection in rat-to-rat passive transfer studies (Phillips et al., 1975; Phillips et al., 1977; Moloney et al., 1987). Humoral responses strongly contribute to rejection of worms in primary and secondary rat infections (Phillips et al., 1975; Cioli et al., 1977; Knopf et al., 1977; Phillips et al., 1977; Cioli et al., 1978; Moloney and Webbe, 1990). This protection could be due to various antibody-mediated killing mechanisms, including IgG and IgE-mediated schistosomula killing by eosinophils and both innate and adaptive complement activation, which have been observed *in vitro* (Ramalho-pinto et al., 1978; Vignali et al., 1988; Moloney and Webbe, 1990; Goes and Ramalho-Pinto, 1991; Miller et al., 1994). Adsorption of IgG2a and IgE from infected rat serum abrogated its protective ability (Ford et al., 1987). Passive transfer of rat hyper-immune serum to mice confers up to 88% protection from challenge. By contrast, transfer of vaccine-immune mouse sera to other mice results in only 20%–62% protection from re-infection, suggesting that the rat is able to mount a more effective humoral response (Mangold and Dean, 1986; Moloney et al., 1987; Moloney and Webbe, 1990; Goes and Ramalho-Pinto, 1991).

All *S. mansoni*-infected hosts examined to date also display an abundant humoral response to parasite glycan antigens (Nyame et al., 2000; Eberl et al., 2001; Naus et al., 2003; van Remoortere et al., 2003; Nyame et al., 2004; Kariuki et al., 2008; van Diepen et al., 2012; Luyai et al., 2014). These antigens include terminal modifications, such as Lewis X (Le^X^), LacdiNAc (LDN), Fucosylated LacdiNAc (LDNF), F-LDN and LDNF-DF, as well as the N-glycan core modifications core α3 fucose (CF) and core xylose (CX) (Nyame et al., 2000; Eberl et al., 2001; Naus et al., 2003; van Remoortere et al., 2003; Nyame et al., 2004; Kariuki et al., 2008; van Diepen et al., 2012; Luyai et al., 2014; Yang et al., 2018; Nkurunungi et al., 2019; Cummings et al., 2022). These glycans also function as immunomodulators and can be recognized by the innate immune system (van Die and Cummings, 2006; van Die and Cummings, 2010; Echeverria et al., 2018; Hambrook and Hanington, 2020; Kuipers et al., 2020; Acharya et al., 2021). Antibodies to schistosome glycans capable of killing schistosomula *in vitro* and protecting mice or rats by passive transfer have been identified by several groups, raising hopes that a well-timed anti-glycan response of the proper magnitude, specificity and isotype composition could be protective in humans (Grzych et al., 1982; Dissous and Capron, 1983; Harn et al., 1984; Grzych et al., 1985; Gregoire et al., 1987; Grzych et al., 1987; Ko et al., 1990; Nyame et al., 2003). However, the *in vivo* role of the anti-glycan component of the immune response has not been well tested in either human populations or protective animal models.

Human N-glycans typically contain a core modification of the GlcNAc residues in GlcNAc-Asn by α6-linked fucose. Core α2-Xylose (CX) and core α3-Fucose (CF) are not found in humans, but are common in N-glycans from insects (CF), plants (CX, CF), and worms (CX, CF) (Khoo et al., 1997; van Die et al., 1999; Altmann, 2007). Expression of CX and/or CF core modifications in schistosomes varies with developmental stage and among species (van Die et al., 1999; Khoo et al., 2001; Smit et al., 2015). In *S. mansoni*, structural and glycomic methods have demonstrated the presence of CX in cercaria, cercarial secretions, young schistosomula and eggs, while CF appears to be restricted to eggs, egg secretions and miracidia (Khoo et al., 2001; Jang-Lee et al., 2007; Smit et al., 2015). Immunologic reactivity to both CX and CF has been demonstrated on *S. mansoni* cercaria, schistosomula, adult worms and eggs (van Die et al., 1999; Faveeuw et al., 2003), suggesting that both of these modifications are present on immunologically vulnerable life stages.

CX and CF appear to be both targets and modulators of the anti-parasite response. Schistosome N-glycans containing CX and CF have been suggested to drive a Th2-biased anti-glycan response in mice. Egg glycoproteins enriched for CX and CF epitopes exert a glycan-dependent Th2-biasing effect on re-stimulated murine T cells, although this was not definitively shown to depend on CX or CF (Faveeuw et al., 2003). Luyai *et al* first demonstrated that schistosome-infected rhesus monkeys and humans generate IgG to CX and CF epitopes, as analyzed on glycan microarrays, with 4/4 monkeys and 4/5 individual humans being reactive (Luyai et al., 2014). The rhesus sera, however, exhibit high titers to CX/CF as well as LDN-based epitopes at the same time points at which they were lethal to schistosomula *in vitro* (Luyai et al., 2014). Brzezicka *et al* also demonstrated that pooled antisera from *S. mansoni* infected children and adults differentially recognized configurations of N-glycan core modifications on a defined glycan microarray (Brzezicka et al., 2015).

Here we characterized the anti-glycan response to *S. mansoni* in brown rats, in contrast to mice, in order to identify factors that might be significant to the differential protection observed in these two hosts. We used glycan microarrays to show that, like humans and rhesus monkeys, rats target CX/CF epitopes during the acute rejection phase of a second infection. We demonstrate that polyclonal antibodies to CX/CF promote complement-dependent and glycan-specific killing of schistosomula *in vitro*. Finally, we determined that antibodies to CX/CF contribute to the schistosomula-lethal activity of rat immune sera *in vitro*. These results suggest that CX/CF is one of the dominant glycan antigens across multiple hosts of *S. mansoni* and that immune responses toward CX/CF could contribute to rejection of schistosomes in resistant animal hosts. The collective role of antibodies to this and other glycan epitopes in human schistosomiasis should be investigated further as potential vaccine targets.

## Results

### S. mansoni-infected rats target CX and CF

As a first step in investigating protective targets of *S. mansoni*-exposed brown rats, we compared rat serum (pooled from animals rejecting a second, high dose infection) to mouse serum for anti-glycan antibodies toward specific glycans in a defined schistosomal array (DSA). Mouse sera from 2, 4, and 6 weeks post infection were from high dose infections and sera from 8 to 20 weeks post infection were from low dose infections, since mice with high dose infections succumb around 8 weeks. The DSA is a collection of structurally defined, semi-synthetic variants of LDN, LDNF, Le^X^, CX and/or CF N-glycans, N-glycopeptides and control glycans, which has been previously validated using lectins, monoclonal antibodies, and infected antisera (Luyai et al., 2014; Prasanphanich et al., 2014) (Supplementary Figure S1).

**FIGURE 1:**
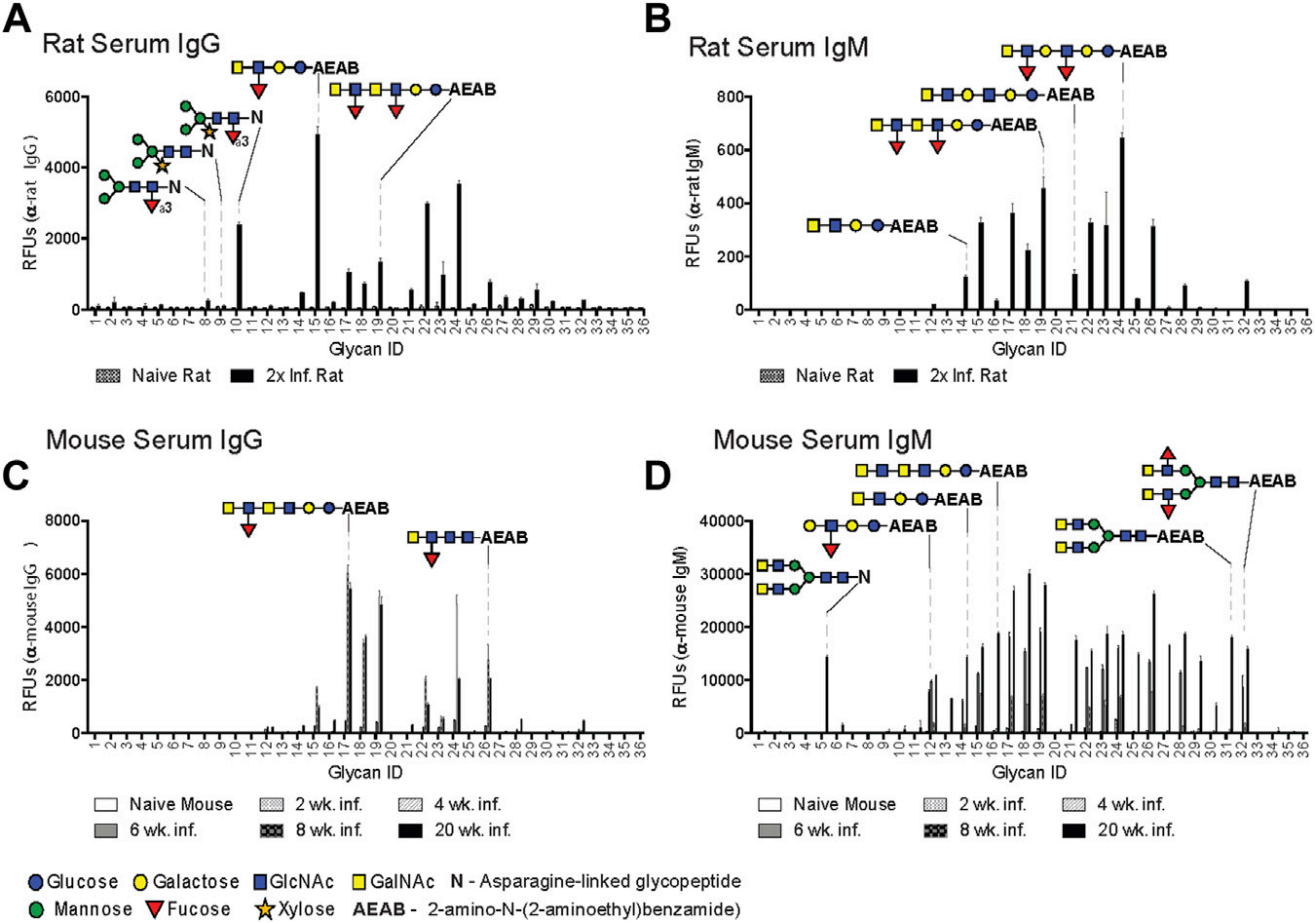
Rats generate IgG to core xylose/core α3 fucose epitope. *Schistosoma mansoni* twice-infected rat serum **(A, B)** and mouse sera from various infection time points **(C, D)**, diluted 1:50 and screened for IgG **(A, C)** and IgM **(B, D)** binding to the Defined Schistosome-type Array (DSA), and detected with goat anti-mouse IgG-Alexa 488, anti-mouse IgM-Alexa 633, goat anti-rat IgG-Alexa 546, and anti-rat IgM-Alexa 488. Each bar represents an average of binding intensity (relative fluorescence units, RFU) to tetra-replicate spots for each glycan ID number (listed on X-axis), +/− 1 standard deviation. Relevant glycan structures depicted, and full list of structures and glycan IDs in Supplementary Figure S1. Glycans are either N-linked (asparagine-linked) or AEAB-linked (bifunctional fluorescent linker, 2-amino-N-(2-aminoethyl)-benzamide); N-glycopeptides contain 1-4 amino acids.

IgG in rat antisera recognized LDNF (glycan ID #15, 17, 19, 22, 24) and, to a lesser extent, LDN (ID #14, 16, 21, 23) variants on the DSA, with the highest IgG binder being a single unit of LDNF (ID #15), and lower binding to extended chain and repeating versions of this antigen (ID #17, 19, 22, 24) (Figure 1A). Interestingly, rat IgG bound highly to CX/CF (ID #10), and preferred the combination epitope to either core xylose (ID #9) or core α3 fucose (ID #8) alone (Figure 1A). The IgM response displayed increased LDN reactivity (ID #14, 21, 23) relative to IgG, but interestingly, no IgM to CX/CF was detected (Figure 1B). Sera from naïve animals had negligible binding.

The mouse anti-glycan IgG response was low through the first 6 weeks after infection and differed in several aspects from that in rats. At 8 and 20 weeks, extended chain variants of LDNF were the primary target (ID #15, 17–19, 22, 24, 26) (Figure 1C). The mouse IgM response was notable for early and sustained targeting of Le^X^ (ID #12) from 4 weeks onward, and broadened to include several biantennary and straight-chain LDN (ID #5, 27–32) and LDNF variants (ID #14–19, 21–26) from weeks 6 through 20 (Figure 1D). Interestingly, no response to CX or CF (ID #8–10) was seen in the mouse antisera from this infection, or during any other mouse infection previously conducted in our laboratory.

The sera were also analyzed for anti-glycan antibodies on the Consortium for Functional Glycomics (CFG) array, a large collection of synthetic, predominantly mammalian-like glycan structures. The CFG structures discussed are depicted in Supplementary Figures S2, S3, and a full listing of printed structures on the CFG version 5.1 can be found at http://www.functionalglycomics.org/static/consortium/resources/resourcecoreh8.shtml. The CFG array does not contain CX/CF since this is not a mammalian glycan. Whereas naïve rat serum demonstrated virtually no binding to the CFG array, IgG from infected rats binds a great breadth of targets, most notably monomeric and polymeric forms of Le^X^ and N-acetyllactosamine (Le^X^: ID#s 70, 154, 292, 419; poly-N-acetyllactosamine: #571–584) (Supplementary Figure S2A). The mouse IgG response was notable for early (4- and 6-week time points) focusing on Le^X^ variants (ID#s 151–154) and LDNF (ID# 97) followed by marked broadening to include several mono- and poly-Le^X^ and N-acetyllactosamine-containing structures, similar to the rat IgG response (Supplementary Figure S2B–E). Serum from chronically infected mice, however, differed from rat serum by more prominent targeting of LDN (ID# 98–99, 528, 563) and LDNF (#97) (Supplementary Figure S2A–E). Similar trends were observed in rat and mouse IgM responses on the CFG array (Supplementary Figure S3A–E).

### Recognition of glycan antigens on glycoproteins

Given that anti-glycan antibodies in rat and mouse antisera recognized many similar (LDN, LDNF, poly-LN) and some distinct (CX/CF) glycan antigens on glycan microarrays, we asked whether their relative targeting of the developmental stages of *S. mansoni* or their relative targeting of glycan *versus* protein antigens differed. Infected mouse serum (8-week) and secondary infected rat serum targeted many of the molecular species in lysates of cercaria, adult worms and eggs on Western blots (Supplementary Figure S4A). A few low molecular weight species in cercaria and adults (15–25 kDa) were notably stained by rat but not by mouse serum, and a 50 kDa species in adult worms was stained by mouse and not rat serum (Supplementary Figure S4A). Mouse serum also exhibited relatively higher intensity staining of soluble egg antigen (SEA) than to earlier life stages, in contrast to rat serum, which targeted cercaria, adults and SEA with relatively equal intensity. Periodate treatment of parasite lysates was used to determine the relative glycan *versus* protein antigen targeting in animal sera. Periodate oxidizes vicinal diols in sugars and destroys their antigenicity. Loss of glycan epitopes upon periodate treatment was confirmed by blotting with AAL (Supplementary Figure S4B). Both rat and mouse sera lost the majority of reactivity with cercarial lysate when it was first periodate treated (Supplementary Figure S4B). This was also confirmed quantitatively by coating treated on ELISA plates. The reactivity of both rat and mouse serum to SEA was reduced by over 50% upon periodate treatment of egg antigen (Supplementary Figure S4C).

Taken together, these data suggest that rats and mice target many of the same molecular species of *S. mansoni*, the majority of which are periodate-sensitive glycans, but with key differences in anti-glycan specificity. Antiserum from twice-infected rats, a naturally resistant host, targets a greater breadth of glycan antigens and prominently targets CX/CF on glycan microarrays. The mouse anti-glycan response, by contrast, is limited during acute infection, broadens only after egg-laying and the chronic immune response are established, and focuses more on LDN and LDNF antigens with notable lack of binding to CX/CF. These findings led us to hypothesize that antibodies to CX/CF might contribute to the natural resistance of rats and other schistosome-infected hosts.

### Rat antisera and rabbit anti-HRP share similar specificity for CX/CF

The glycopeptides used to prepare the CX and CF-containing structures on the DSA were derived and modified chemo/enzymatically using the plant glycoprotein HRP as a resource, as it possesses N-glycans that are about 80% core α2-xylosylated and core α3-fucosylated (Yang et al., 1996). The reactivity of animal antisera with this epitope on glycan microarrays suggests that the *S. mansoni* core epitope, which provokes the response in mammals, is cross reactive with CX/CF moieties on HRP glycoprotein. Since it is difficult to isolate the contribution of just one antibody specificity in a complex humoral response to the parasite, we exploited rabbit anti-horseradish peroxidase (rabαHRP), a commercially-available polyclonal antibody to HRP, as a proxy. It was previously demonstrated that the specificity of rabαHRP is primarily directed at the CX/CF antigens within HRP N-glycans *via* cross-reactivity with other well-defined plant glycoproteins and inhibition by neo-glycoconjugates (Faye et al., 1993; Faveeuw et al., 2003). We verified this by demonstrating near-complete loss of reactivity of rabαHRP binding to HRP-coated ELISA plates treated with periodate (Supplementary Figure S5A). Additionally, rabαHRP displays minimal binding to other glycan structures on the CFG array (data available on version 4.2 for anti-HRP antibody from Sigma, at http://www.functionalglycomics.org/glycomics/publicdata/selectedScreens.jsp), and those which are bound are not expected to be found in *S. mansoni*, validating its utility to mimic the CX/CF specificities found in rat antisera.

**FIGURE 2:**
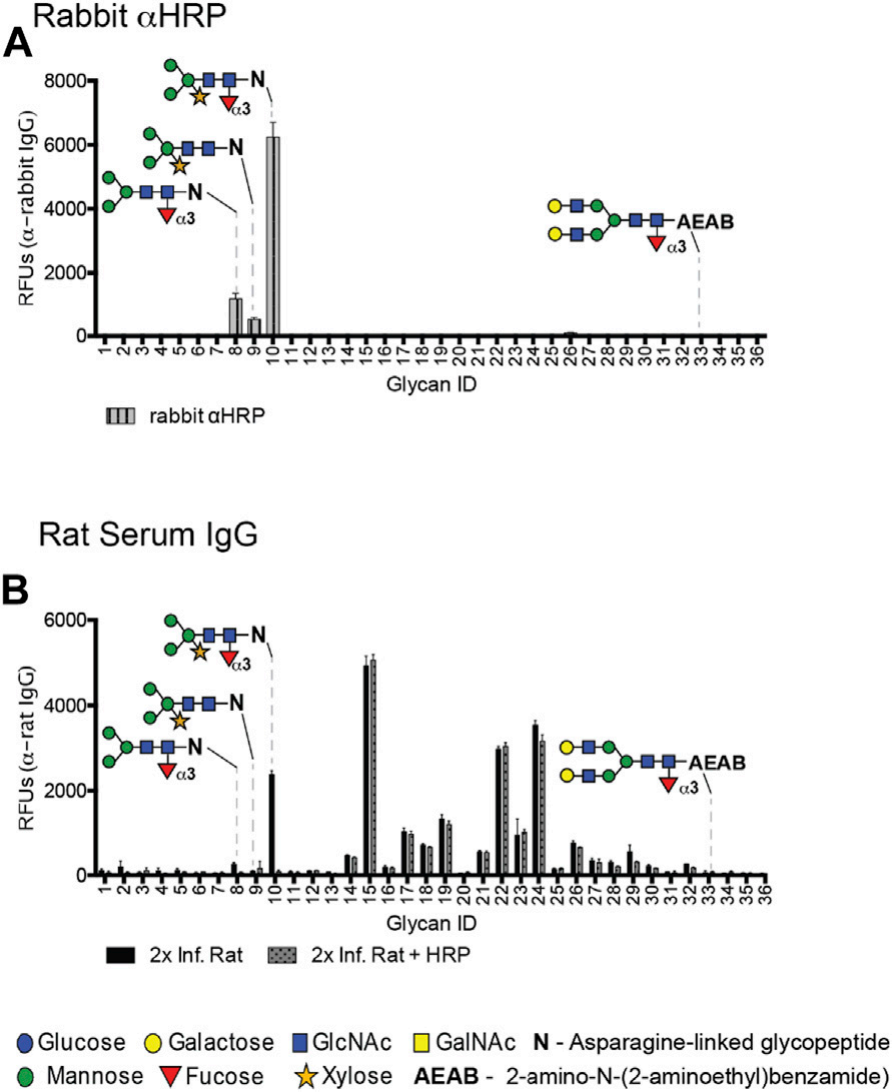
Rat antisera and rabbit anti-HRP share similar specificity for CX/CF on the DSA. **(A)** Rabbit anti-HRP screened on the DSA at 20 μg/mL and detected with goat anti-rabbit IgG-Alexa 488. **(B)** Rat serum at 1:50 screened for IgG on the DSA with or without pre-incubation with 100 µg HRP in the binding buffer.

To compare the specificity of rabαHRP with rat serum, we utilized glycan microarrays and Western blots of several well-characterized plant glycoproteins. RabαHRP has similar core epitope specificity when compared with rat antisera on the DSA, preferring the dual CX/CF epitope (ID #10) over structures containing CF or CX alone (ID #8, 9, 33) (Figures 2A,B). On Western blots, both rat antisera and rabαHRP displayed the highest reactivity with HRP, followed by phytohemagglutinin (PHA), which both contain the dual CX/CF epitope. RabαHRP was also faintly reactive with bromelain (BRO, containing CX on a truncated bi-mannose core) (Supplementary Figure S5B). Both modalities demonstrate that rabαHRP mimics the propensity of rat antisera for the dual CX/CF epitope. This could be due to an additive effect of monoclonal fractions specific for CX and CF, or a true clonal preference for the conformation of the dual epitope. Therefore, we considered rabαHRP a useful proxy for further investigation of the anti-CX/CF response in schistosome infection. Throughout the remainder of the manuscript, we refer to anti-CX/CF reactivity in the rabbit polyclonal and rat antisera, with the understanding that it may comprise fractions directed either at CX, CF or the dual epitope. Given this close overlap in specificity, we then demonstrated that pre-incubation of rat antisera with HRP completely abrogated binding to CX/CF (ID #10) while other anti-glycan reactivities in the rat serum remained intact (Figure 2B). These results demonstrate that HRP and other CX/CF-containing glycoproteins are useful tools to specifically isolate anti-glycan reactivities in animal sera.

### Rabbit anti-HRP is lethal to schistosomula

**FIGURE 3:**
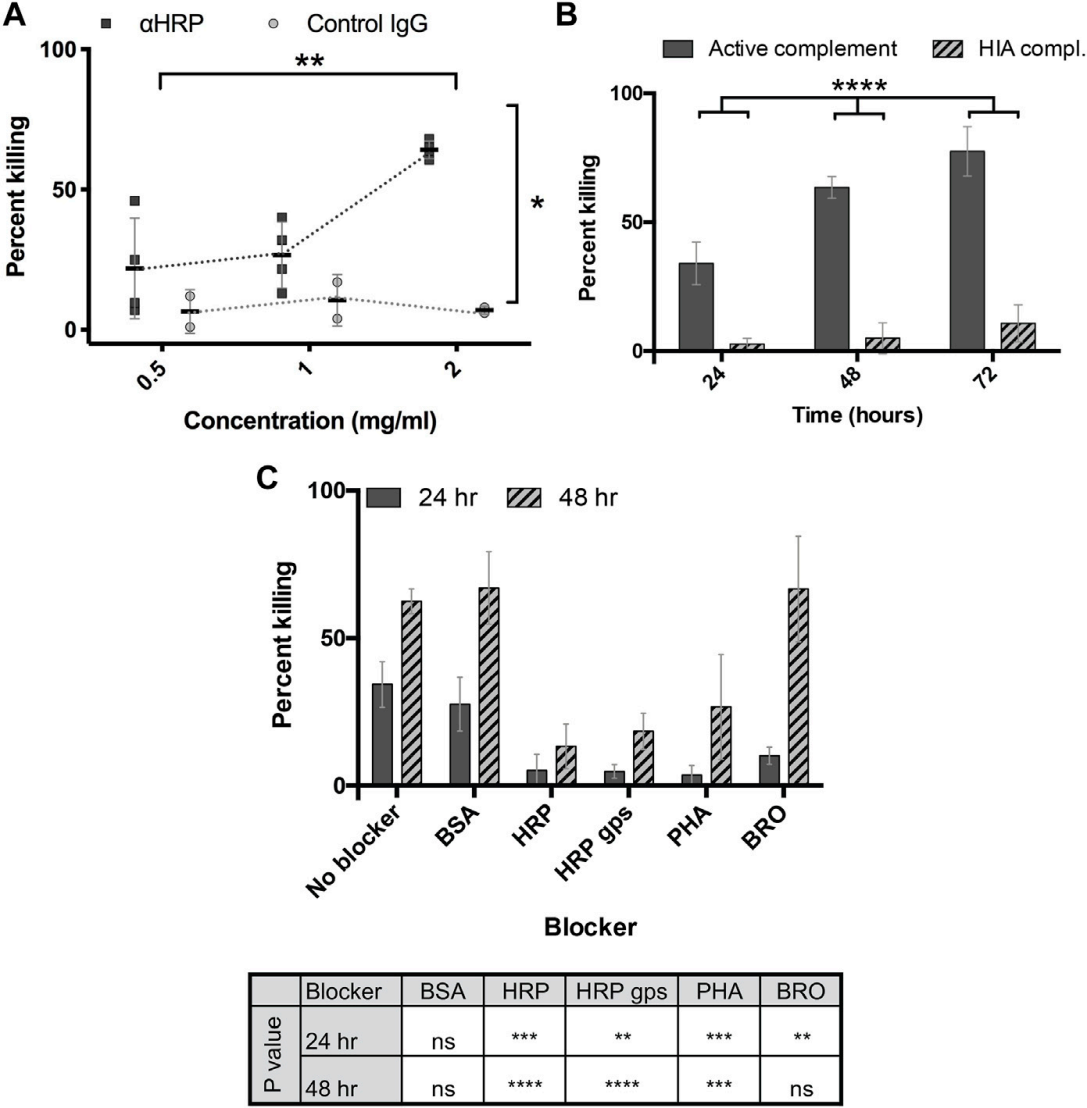
Anti-HRP kills schistosomula *in vitro.* **(A)** Rabbit anti-HRP and normal rabbit IgG at indicated doses incubated with 3h transformed schistosomula and active guinea pig complement, and percentage of non-viable out of total schistosomula quantified at 48 h. 2-way repeated measures ANOVA showed a significant effect of antibody type and dose. *p < 0.05; **p < 0.01. **(B)** Rabbit anti-HRP at 2 mg/mL incubated with 3h transformed schistosomula and active or heat-inactivated (HIA) guinea pig complement, and percentage of non-viable out of total schistosomula quantified at 24–72 h. 2-way repeated measures ANOVA showed a significant effect of time and complement activity. ****p < 0.0001. **(C)** Rabbit anti-HRP at 2 mg/mL, pre-incubated with or without the indicated blockers (50 μg per well), incubated with 3h transformed schistosomula and active guinea pig complement, and percentage of non-viable out of total schistosomula quantified at 24–48 h. 2-way repeated measures ANOVA with Sidak’s multiple comparisons test showed a significant effect of time and blocker, with the level of significance for each blocker compared to control shown in the table. ns, not significant; **p < 0.01, ***p < 0.001 ****p < 0.0001. BSA, Bovine serum albumin, HRP, Horseradish peroxidase, HRP gps, HRP glycopeptides, PHA, phytohemagglutinin-E, BRO, Pineapple bromelain.

Given that brown rats exert significant anti-schistosomal activity during the early weeks of primary infection and then rapidly eradicate a second infection (Knopf et al., 1977; Cioli et al., 1978; Sepulveda et al., 2010), we asked whether antibodies to CX/CF had activity against schistosomula *in vitro*. RabαHRP indeed killed 3-h old schistosomula at a significantly higher rate than the negative control (normal rabbit IgG) in an *in vitro* complement-mediated killing assay (*p* < 0.05) (Figure 3A). Killing by rabαHRP was dependent upon concentration of the antibody (Figure 3A) (*p* < 0.01) and the presence of active guinea pig complement (*p* < 0.0001) (Figure 3B). To investigate whether killing depended on anti-glycan targeting, glycoproteins were used as blocking agents. Killing of schistosomula by rabαHRP was not inhibited by BSA (an irrelevant non-glycosylated protein), but was significantly inhibited by the glycoproteins HRP, PHA and to a lesser extent, BRO (Figure 3C). In order to determine if killing activity was truly dependent on the glycan epitopes targeted by rabαHRP, we prepared glycopeptides *via* Pronase digestion of HRP, which in a limit digestion produces glycopeptides containing Asn and few if any other amino acids. Glycopeptides were verified to be 80% glycan by weight, the highest abundance of which matched a composition of Hex3HexNAc2Fuc1Xyl1-NR, as already demonstrated by Song *et al.* (Song et al., 2009). HRP glycopeptides significantly inhibited rabαHRP killing activity, down to the level seen with the same concentration of control rabbit IgG (Figure 3C). Based on the known glycan targeting of rabαHRP and the ability of both cross reactive glycoproteins and Pronase-digested HRP glycopeptides to inhibit schistosomula killing, we conclude that antibodies to CX/CF are capable of killing schistosomula *in vitro* in a complement-dependent and glycan-specific manner.

### CX/CF is expressed on surface and secretions of S. mansoni larval life stages

**FIGURE 4:**
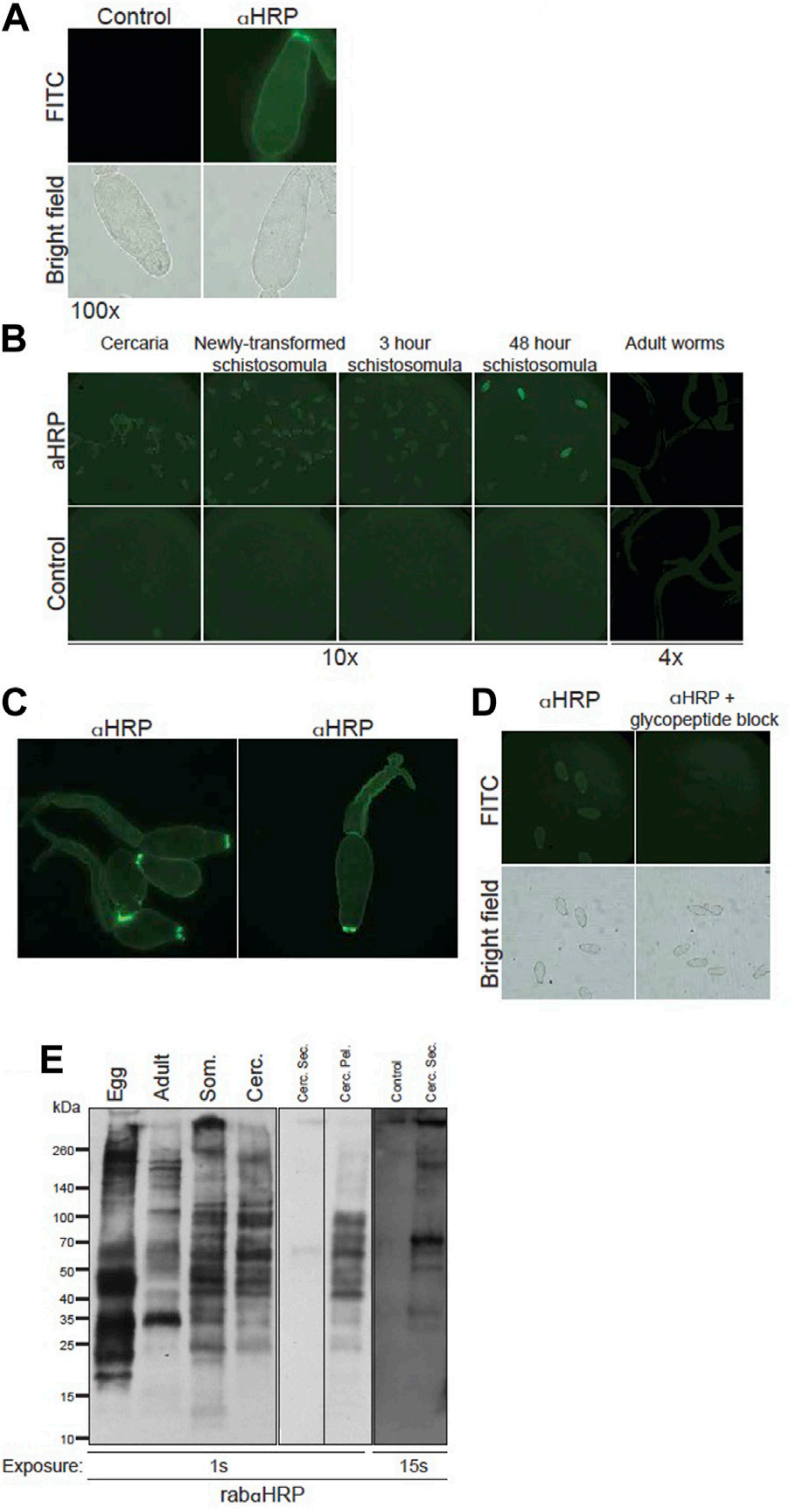
Core xylose/core fucose expressed on surface and secretions of *S. mansoni* larval life stages. **(A)** Fixed *S. mansoni* cercaria stained with 100 μg/mL anti-HRP or control (normal rabbit IgG), anti-rabbit-Alexa488, mounted on slides and visualized at 100x (exposure time 207.8 ms, FITC 539.4 ms). **(B)** *S. mansoni* cercaria, *in vitro* isolated schistosomula, and murine-isolated adult worms stained with rabbit anti-HRP or control (normal rabbit IgG) and visualized in 96-well plates; larvae imaged at 10x (exp time 1.051 s, FITC 490.3 ms), adults imaged at 4x (exp time 6.115 s, FITC 717.9 ms). **(C)** Cercaria stained with anti-HRP and imaged at 20x using deconvolution microscopy. **(D)** 3 h schistosomula stained with anti-HRP, (left), or anti-HRP pre-incubated with HRP glycopeptides (right) and imaged at 20x (exp time 1.051 s, FITC 490.3 ms). **(E)** Western blot of rabbit anti-HRP staining: *S. mansoni* soluble egg antigen (Egg), RIPA extracts of adult worms (Adult), 3 h schistosomula (Som) and whole cercaria (Cerc) each with 8 μg loaded per well (left panel); Concentrated supernatant of cercarial secretions (Cerc. Sec., 1.4 μg), secretion-depleted cercarial pellet (Cerc. Pel., 6.7 μg) (middle panel); aquarium water concentrate as negative control (control), cercarial secretions (1.4 μg) and secretion-depleted cercarial pellet (6.7 μg) (right panel) at indicated exposure times.

Protection exhibited by naturally resistant animal hosts may depend on immune activity against multiple schistosome life stages, and many glycoprotein antigens are known to be up- or downregulated as the worm matures. We therefore analyzed the developmental expression and epitope distribution of CX/CF on intra-mammalian life stages *via* immunofluorescence and Western blotting. RabαHRP, but not control rabbit IgG, robustly stained cercaria (Figure 4A). Interestingly, staining was brightest around the oral sucker of the cercarial head, and this persisted through at least 3 h post transformation (Figures 4A,B). Cercarial heads, tails, and schistosomula through 48 h post transformation showed approximately equal abundance of staining by rabαHRP (Figure 4B). Intact, fixed adult worms, however, did not stain beyond the background level seen with control antibody (Figure 4B). To get a better idea of the exact distribution of CX/CF epitopes, we imaged cercaria using deconvolution microscopy (Figure 4C). Staining was concentrated in the tegument surrounding the entire organism, slightly greater than that seen in internal tissues. The brightest areas on the tip of the oral sucker appear to localize to the lateral crescents and/or the apertures of the penetration gland ducts (Ishikawa et al., 1970). Staining of 3-h schistosomula was blocked by addition of Pronase-digested HRP glycopeptides, demonstrating that the staining is specific for glycan epitopes (Figure 4D).

Western blotting confirmed that CX/CF epitopes are expressed throughout the larval life stages of *S. mansoni* as well as on adult worms and SEA (Figure 4E). The banding patterns on cercaria and schistosomula were very similar, whereas banding patterns on adult worms and SEA were each distinct. Given the concentrated staining of the cercarial oral sucker observed with immunofluorescence, we prepared cercarial secretions and a corresponding secretion-depleted cercarial pellet. Cercarial secretions had substantially less protein content than pellet, and had to be serially concentrated. A sample of aquarium water was serially concentrated in parallel to control for potential contaminants from the non-sterile snail habitat. In spite of serial concentration, the maximal amount of cercarial secretions that could be loaded for SDS-PAGE was about 25% of the amount of protein loaded for the cercarial pellet. Multiple molecular species in cercarial secretions were immuno-reactive for CX/CF, whereas the aquarium water control did not stain even at higher exposure times (Figure 4E). The pellet was robustly stained by rabαHRP, and comparable in magnitude and banding pattern to the staining of whole cercarial lysate. Although the variable amount of protein loaded precludes quantitative comparison of staining on the cercarial secretions *versus* pellet, CX/CF is clearly present on distinct molecular species in the secretions compared to the pellet. From these studies, we conclude that CX/CF is abundantly expressed throughout the intra-mammalian life stages of *S. mansoni*, on a variety of molecular species which vary as the parasite develops. Importantly, CX/CF is expressed on the surface of vulnerable life stages of the parasite (cercaria and schistosomula), and on both somatic and secreted antigens of cercaria, providing accessible targets for attack by destructive antibodies *in vivo*.

To isolate schistosomula glycoproteins carrying this important glycan epitope, we conjugated rabαHRP to Ultralink beads and immunoprecipitated glycoproteins from 3 h schistosomula. Several glycoproteins were pulled out from sizes 25–45 kDa, as well as smaller amounts of species from 70 to 100 kDa, around 140 kDa and above 260 kDa (Supplementary Figure S6). These species were not isolated by control beads (conjugated with normal rabbit IgG), and could be re-stained with rabαHRP, indicating the specificity of the immunoprecipitation. Unfortunately, insufficient material was obtained to pursue molecular identification of the precipitated schistosomula glycoproteins *via* proteomics. Given the previously demonstrated specificity in the literature (Faye et al., 1993; Faveeuw et al., 2003) and re-demonstrated in our studies (Supplementary Figure S5A) of rabαHRP for CX/CF, rather than protein epitopes, this data shows that glycan epitopes can be utilized as immunoprecipitation targets.

### Antibodies to CX/CF contribute to schistosomula killing in rat serum and rhesus serum

**FIGURE 5:**
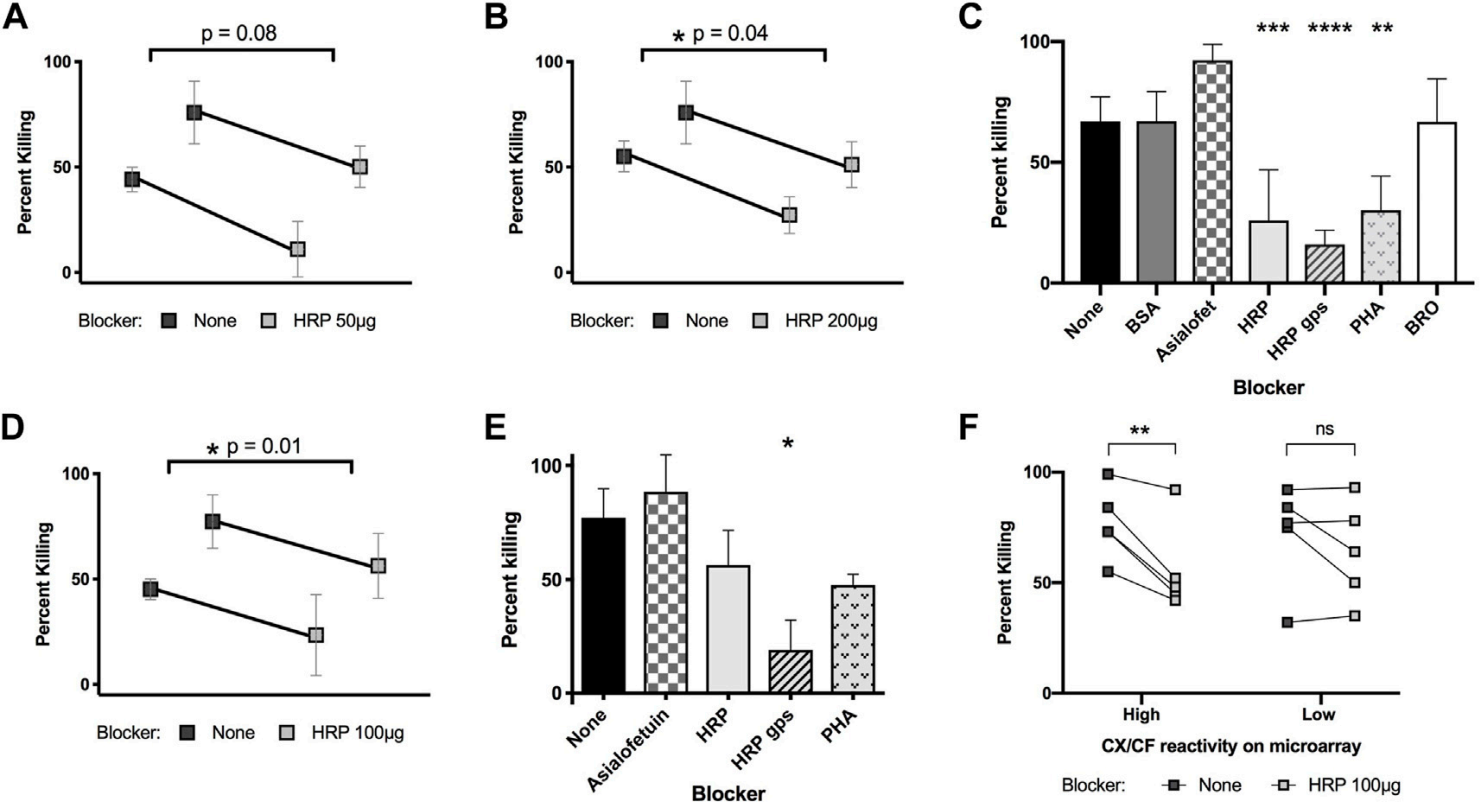
Killing of schistosomula by infected rat and rhesus serum with glycoprotein blockers. 3 h schistosomula were incubated with active guinea pig complement and pooled rat serum **(A–C)** or rhesus serum **(D, E)** from secondary infection, which had been pre-incubated with the indicated blocking glycoproteins or glycopeptides. Percentage of non-viable out of total schistosomula were quantified at 48h. Paired t-tests were performed for sera with or without HRP blocking with the indicated *p*-values **(A,B, and D)**. In **(C, E)**, concentrations of blocker are 50 μg (asialofetuin, BSA, HRP, PHA, BRO) or 25 μg (HRP glycopeptides). One-way ANOVA with Dunnett’s multiple comparisons was performed to compare each blocker to control (no blocker) *p < 0.05; **p < 0.01, ***p < 0.001 ****p < 0.0001. In **(F),** 10 samples from individual infected rhesus monkeys were stratified by high (>1,000 RFU) or low (≤1,000 RFU) reactivity to CX/CF epitope on the DSA, and tested for schistosomula killing with or without HRP blocking (100 μg/well). 2-way ANOVA with Sidak’s multiple comparisons showed significant effect of blocking for rhesus samples with high CX/CF reactivity, but not for blocking of samples with low CX/CF reactivity.

Knowing that rabαHRP is capable of killing schistosomula *in vitro*, and that this is only one of many anti-glycan and anti-protein specificities found among the antibodies of animals responding to *S. mansoni* infection, we asked to what extent antibodies to CX/CF were contributing to the killing ability of infection antisera by blocking with HRP and other glycoproteins or glycopeptides. Similar to infected rat serum, previous studies by our group also found that rhesus serum binds CX/CF on glycan arrays (Luyai et al., 2014). Both pooled hyper-immune rat serum (Figures 5A–C) and pooled rhesus infection antisera (Figures 5D,E) killed schistosomula *in vitro* in a complement dependent manner. Interestingly, the killing activity of both rat and rhesus pooled antisera could be partially inhibited by blocking with HRP (Figures 5A,B,D) but not by blocking with irrelevant glycoproteins BSA and asialofetuin (Figures 5C,E). Similar to what was seen when blocking rabαHRP killing, HRP glycopeptides were the most potent inhibitor, with HRP and PHA glycoproteins exerting a less pronounced effect (Figures 5C,E). To control for any non-epitope-specific effect of HRP on serum-mediated killing, we compared the ability of HRP to block killing by individual rhesus sera that were stratified by high titer *versus* low titer antibodies to CX/CF based on their DSA binding. HRP significantly blocked killing only in antisera with high titer antibodies to CX/CF and did not inhibit killing of antisera which lacked this specificity (Figure 5F). This novel data demonstrates that antibodies to CX/CF are major contributors to *in vitro* schistosomula killing activity by both rat and rhesus antisera.

Given the impressive ability of CX/CF-containing glycoproteins and glycopeptides to block the schistosomula killing activity of infected animal sera, we screened sera from many individual rhesus monkeys responding to schistosome infection at various time points to determine if there was a correlation between levels of antibody to CX/CF on glycan microarray and killing ability *in vitro*. Rhesus monkeys displayed a highly variable response to CX/CF and other glycan epitopes on the DSA throughout the course of infection (Supplementary Figure S7). The magnitude of response to CX/CF on glycan microarray showed only a weakly positive and non-significant correlation with the extent of schistosomula killing (Supplementary Figure S7). Interestingly, however, we saw moderate and highly significant correlations between level of IgG and IgM to several of the array antigens terminating in Le^X^, LDN, and LDNF motifs and schistosomula killing by individual rhesus sera at 48 h (Supplementary Figure S7). Thus, although CX/CF-specific antibodies are significant contributors to schistosomula killing *in vitro,* there are likely many other glycan specificities and aspects of the humoral immune response which combine in a complex manner to eliminate schistosomula.

## Discussion

The aim of this study was to examine the anti-glycan humoral immune response to *S. mansoni* in a naturally resistant model, the brown rat. We and others (Omer-Ali et al., 1986; Omer-Ali et al., 1989; Eberl et al., 2001; Kariuki et al., 2008) have previously observed that antisera from both twice-infected rats and chronically infected mouse were primarily directed towards glycan antigens of *S. mansoni* cercaria and SEA, as evidenced by the disappearance of broad reactivity with periodate-treated antigens and robust anti-glycan responses on glycan microarrays. Thus, we hypothesized that differences in anti-glycan specificity may play a key role in the differential natural resistance between these two rodent hosts. Our key finding was that although rats target many of the same glycan specificities as mice, they also differ significantly from mice in producing robust levels of IgG to core xylose and core α3 fucose (CX/CF) epitopes, which prompted us to further investigate the role of such antibodies in the anti-schistosomal response. We further observed that antibodies targeting CX/CF, both as a polyclonal preparation and within the antisera of infected animals, kill schistosomula *in vitro*, suggesting that these antibodies may contribute to a protective anti-schistosome response *in vivo*.

Our novel observation that rats, as has previously been shown for rhesus monkeys and humans (Luyai et al., 2014; Brzezicka et al., 2015; Yang et al., 2018), produce IgG to CX and CF epitopes, suggests that these are dominant epitopes in the anti-schistosomal response across multiple hosts. We exploited the curious cross reactivity of schistosome CX/CF epitopes with the plant glycoprotein HRP, and a commercial rabbit polyclonal antibody to HRP, to better understand the expression of this epitope on *S. mansoni* life stages and assess the role of rat antibodies targeting CX/CF. Immunofluorescence studies and Western blots demonstrated abundant expression of CX/CF containing glycoproteins throughout all of the intra-mammalian life stages of *S. mansoni*, including on cercaria and early schistosomula, which concentrated at the cercarial oral sucker, and was not abrogated by removal of cercarial secreted products. In agreement with our data, expression of this epitope has been shown on intramammalian stages of *S. mansoni*, particularly in the post-acetabular glands of schistosomula and in the cecal epithelium and sexual organs of adult worms (Faveeuw et al., 2003). This suggests that CX/CF epitopes are expressed on somatic antigens of the parasite larvae, and are accessible targets of the immune system during early infection.

An important future direction for exploitation of this epitope in vaccine development will be to determine exactly to which epitope or combinations of epitopes in schistosomes—CX, CF or the dual CX/CF epitope—infected rats, rhesus and humans respond, and precisely which antibody specificities are most lethally potent against schistosomula. All studies thus far have used polyclonal antisera or antibodies, and to answer this question may require development of individual monoclonal reagents. Immunization of mammals with plant glycoproteins carrying the dual epitope (both CX and CF) causes an antibody response which can be fractionated by reactivity with either CX or CF, suggesting they form two distinct epitopes (Faye et al., 1993). We observed *via* Western blot and glycan microarray that both polyclonal rat anti-sera and rabαHRP were most reactive with the dual epitope (glycan ID #10) and glycoproteins containing the dual epitopes (HRP and PHA), compared with the two glycans containing either CF or CX alone (glycan ID #8–9) or BRO which contains only CX. This preference might indicate antibodies that truly are specific for the dual epitope, or separate CX- and CF-reactive fractions which still prefer the epitopes in the dual-configuration. Co-crystallization of such a monoclonal antibody, if obtainable, to these epitopes might help to address that issue.

MALDI-TOF-MS data initially suggested that *Schistosoma japonicum* eggs contained CX, core α3-and α6-fucose on the same glycans while *S. mansoni* egg glycans contained only CX and core α6-fucose (Khoo et al., 1997). Smit *et al.* demonstrated the presence of the dual epitope in PNGase-A-released N-glycans from *S. mansoni* eggs (Smit et al., 2015)*.* However, rats, because of their development of resistance, have very little exposure to mature eggs. Structural and glycomic methods have demonstrated CX in cercaria, cercarial secretions and young schistosomula, disappearing around 1 week post infection (Khoo et al., 1997; Khoo et al., 2001; Jang-Lee et al., 2007; Smit et al., 2015). Thus, one hypothesis is that rats primarily respond to CX during the early stages of infection, but that these antibodies prefer the CX epitope in the structural configuration of N-glycans bearing CX/CF. Immunologic reactivity to both CX and CF has been previously demonstrated on *S. mansoni* schistosomula and adult worms, in addition to eggs (van Die et al., 1999; Faveeuw et al., 2003). Therefore, it is also possible that rats are responding to small but highly antigenic amounts of the dual epitope on early stages of *S. mansoni* or on relatively small amounts of egg antigens later in infection. Luyai *et al.* showed that rhesus monkeys and newly exposed humans also reacted most strongly to the dual antigen but bound significantly to CF alone and less so to CX alone, and that some humans preferred CX alone to the dual epitope (Luyai et al., 2014). This seems credible given the increased exposure to eggs in humans and rhesus monkeys compared to rats.

Another interesting hypothesis would be that greater targeting of CX, which appears to be most abundant in the early life stages, would result in increased resistance in rhesus monkeys or humans. This is supported by Brzezicka *et al.*, who showed that sera from adults in endemic areas, who generally exhibit “partial resistance,” bound glycans with CX or CX/CF but not CF alone, as opposed to children, who responded to almost all of the accessible CX or CF-containing glycans (Brzezicka et al., 2015). Nkurunungi *et al.* also reported IgE and IgG to CX positively associated with *S. mansoni* infection and with infection intensity (Nkurunungi et al., 2019). However, whether the correlation of CX antibodies with infection in humans extends to a protective role for these antibodies remains to be determined.

Why do rats target CX and CF in response to *S. mansoni* infection, while the mice in our study do not? This is an interesting question, as these finding has been replicated over numerous infections of Swiss Webster mice in our hands (Luyai et al., 2014; Prasanphanich et al., 2014). Other investigators have noted that immunization of certain strains of mice with HRP fails to produce glycan-specific antibodies to the core epitopes, while immunization of rats and rabbits with HRP does induce a glycan-specific response (Bardor et al., 2003; Jin et al., 2006; Hino et al., 2010). These differences in host responses could be related to differences in the character of immunity generated by different hosts to the parasite, genetic differences or tolerization of the mouse to certain epitopes. C57BL/6 were reported to produce glycan-specific antibodies in response to HRP immunization, whereas BALB/c did not (Bardor et al., 2003; Faveeuw et al., 2003). However, Swiss-Webster mice are outbred, making it unlikely that genetic differences would be the reason for their lack of response in our study. The *S. mansoni*-infected C57BL/6 used in Faveeuw *et al.* made a weak but detectable IgG1 response (Th2-restricted) to plant glycoproteins carrying CX and CF (Faveeuw et al., 2003). Thus, for unclear reasons, some mice do make a glycan-specific response to CX and CF in schistosome infection, while others do not. These strains all develop chronic infection, suggesting the immune factors contributing to clearance in mice are not limited solely to differences in glycan antibody specificity. Direct comparison of resistance to schistosomiasis primary infection and re-infection or response to vaccination among murine strains which either can or cannot target CX/CF, and among genetically identical mice with passively transferred antibodies, are key next steps to elucidating the role of these antibodies.

A great breadth of anti-glycan specificities was observed in rat antiserum on the CFG array, including Le^X^-containing glycans among the highest binders and lower-level binding to many poly-LN containing structures. Mice display relatively sparse anti-glycan targeting early in infection, also focused on Le^X^ epitopes, and develop a breadth comparable to rats only after egg-laying has commenced, which targets Le^X^, LN, LDN and LDNF. Another group recently reported on the glycan binding of low-to medium-dose infected rat antibody-secreting cell probes, which also identified Le^X^ and Poly-LNs as two of the major motifs recognized, along with β3-GlcNAc terminating structures (McWilliam et al., 2013). Le^X^, LN and polymeric variants LN are also found in the mammalian glycome (reviewed in (Cummings, 2009)). However, this does not rule out the possibility that they could be presented on the parasite in a unique fashion and/or be effective targets of anti-parasite immunity. One hypothesis is that having many anti-glycan specificities, or many anti-glycan antibodies with relatively low affinity/specificity, could be advantageous for binding determinants that are common and densely distributed on the parasite. Why twice-infected rats produce a broader response than mice during a primary infection could be related to differences in amount or duration of exposure to egg antigens, or differences in tolerization to self-similar antigens in these two hosts. Another hypothesis is, given that many of the glycan antigens targeted by rats and mice are the same (Le^X^, LDN, LDNF), perhaps the mouse possesses some inhibitory factor, such as blocking antibodies, which are not present in rats. Blocking antibodies have been observed in rats, mice and hypothesized to exist in endemic humans (Grzych et al., 1984; Omer-Ali et al., 1986; Butterworth et al., 1987; Dunne et al., 1987; Dunne et al., 1992). Other immune factors such as IL-10 have also been observed to block the development of resistance to re-infection in mice (Wilson et al., 2011).

Complement-mediated lysis of schistosomula and antibody-dependent cellular cytotoxicity have both been demonstrated using rat serum or rat monoclonal antibodies *in vitro,* and appear to be important *in vivo* (Phillips et al., 1975; Ramalho-pinto et al., 1978; Capron et al., 1981; Grzych et al., 1982; Vignali et al., 1988). Antibodies to glycans could be involved in such protective mechanisms. To assess the prospect of CX/CF as a protective epitope, we showed that antibodies to CX/CF, both polyclonal rabbit anti-HRP and antibodies targeting this specificity in rat and rhesus antisera, kill schistosomula *in vitro* in a complement-dependent and glycan-specific fashion. This in combination with immunofluorescence studies suggests that antibodies to CX/CF are able to bind the schistosomula surface and fix complement in sufficient quantity so as to disrupt its integrity and result in death. Relatively high concentrations (0.5–2 mg/mL) of antibody were required for killing. Our previous studies and unpublished data have demonstrated that other anti-glycan antibodies are able to kill schistosomula at concentrations as low as 0.05 mg/mL (IgG) (Nyame et al., 2003; Mickum et al., 2016). This could be related to differences in the density and distribution (i.e., in key locations of weakness for the parasite) of different glycan epitopes. Luyai *et al.* also showed that the killing ability of rhesus antisera correlated with the overall anti-glycan titer at various time points during infection, but this study was of one individual monkey (Luyai et al., 2014). Yang *et al.* similarly observed that 22-week rhesus antisera killed schistosomula *in vitro* and hypothesized that antibodies to multi-fucosylated LDN motifs may contribute to killing, due to their persistence at this time point, while other anti-glycan responses were decreasing (Yang et al., 2017). Using a panel of sera from many individual rhesus monkey at different infection time points, we saw that the titer to CX/CF does not correlate strongly with schistosomula killing ability, but the titer of IgM and IgG to several other glycan epitopes including LDN and LDNF does significantly correlate with killing ability. We hypothesize that *in vivo*, antibodies to multiple and diverse glycan and protein epitopes work additively for efficient killing of schistosomula. Passive transfer of monoclonal anti-glycan antibodies to susceptible animal hosts could better assess their protective capacity *in vivo* in future studies.

A few small glycoproteins ≤35 kDa in cercarial and adult worm lysates were targeted by both rat serum and rabαHRP, but not mouse serum, suggesting that rats may target CX/CF on some of the same parasite glycoprotein species targeted by rabαHRP. Rat serum reactivity to these species is abolished by periodate treatment, confirming the glycan target of the antibodies. The identities of the CX/CF-bearing glycoprotein species are of great interest, given their targeting by multiple hosts (Luyai et al., 2014). Interestingly, two of these bands correspond roughly in size (13 kDa, 35 kDa) to glycoproteins identified by our collaborators and others as members of the SmLy6 family, which are *S. mansoni* homologs of the human complement inhibitory factor CD59, although their function as complement inhibitors in schistosomes has been called into question (Sepulveda et al., 2010; Farias et al., 2013; Chalmers et al., 2015; Krautz-Peterson et al., 2017). Members of this family such as Sm29 are present on the *S. mansoni* tegumental surface, have probable glycosylation sites, and are vaccine candidates in pre-clinical evaluation (Cardoso et al., 2006a; Cardoso et al., 2006b; Cardoso et al., 2008; Castro-Borges et al., 2011; Farias et al., 2011; Farias et al., 2013; McWilliam et al., 2014; Pinheiro et al., 2014; Gonçalves de Assis et al., 2015). We performed immunoprecipitation of the CX/CF-containing glycoproteins from schistosomula lysates, which pulled out a handful of molecular species between 25–45 kDa and 70–100 kDa. Molecular identification of these species from vulnerable schistosome life stages, and characterization of their stage-specific, native glycosylation, are crucial next steps to their assessment as vaccine candidates.

In spite of several decades of research, only a few vaccine candidates for schistosomiasis have progressed to human testing and none are currently licensed (McManus and Loukas, 2008; Kupferschmidt, 2013). Unfortunately, while the utility of schistosome glycans as candidate vaccine epitopes has been long proposed, their potential as vaccines has not been explored in depth. One reason for the general failure of anti-schistosomal vaccines may be that the vast majority of candidates are identified and produced using protein-focused screening and recombinant production methods, which overlook the importance and uniqueness of glycosylation in this parasite (Braschi and Wilson, 2006; Hotez et al., 2010; Mulvenna et al., 2010; Gaze et al., 2014; McWilliam et al., 2014; Crosnier et al., 2019; Neves et al., 2020). Based on the current studies here, and prior studies in the field on this subject, glycan-based vaccines might need to be reconsidered. While there has been some concern that CX/CF epitopes represent potential “cross-reactive carbohydrate determinants” targeted by IgE, as seen in allergic patients, 25%–50% of non-allergic donors also harbor such antibodies, and several studies have demonstrated that CX and CF are not clinically significant in allergic individuals (Altmann, 2007; Hoffmann-Sommergruber et al., 2011; Altmann, 2016). Recent studies clearly identify IgE to the CX and CF epitopes in Ghanaian children, which may also be cross-reactive (Amoah et al., 2018). Additionally, schistosomiasis-infected individuals have a downregulated allergic response (reviewed in (Fallon and Mangan, 2007)). Thus, these epitopes should not be discounted as possible vaccine antigens based solely on the finding that they are potential targets of human IgE. It is likely that the protein carrier, valency of the epitope, and other innate immune cues provided by the parasite affect the character of immunity generated to specific antigens, which should be further investigated (Altmann, 2007). An ideal vaccine candidate would likely include *both* glycan and protein epitopes which are exposed on the outer and/or mucosal surfaces of the parasite, expressed early during infection and sustained, and should generate a more robust humoral response that is toxic to both schistosomula and adult worms. To achieve robust, multi-pronged immunity against this complex parasite, glycomics strategies should be married with other novel -omics approaches, which have pointed to several new vaccine candidates based on surface accessibility and stage-specific local immune response (Cardoso et al., 2006a; Cardoso et al., 2006b; Braschi et al., 2006; Braschi and Wilson, 2006; Fallon and Mangan, 2007; Cardoso et al., 2008; McManus and Loukas, 2008; Driguez et al., 2010; Mulvenna et al., 2010; Castro-Borges et al., 2011; Farias et al., 2011; Hoffmann-Sommergruber et al., 2011; Loukas et al., 2011; McWilliam et al., 2012; Kupferschmidt, 2013; Gaze et al., 2014; McWilliam et al., 2014; Pinheiro et al., 2014; Altmann, 2016; Sotillo et al., 2016; Amoah et al., 2018; Crosnier et al., 2019; Neves et al., 2020).

In conclusion, we identified robust targeting of CX/CF in the anti-glycan response of rats and found that antibodies targeting this epitope kill schistosomula *in vitro*. The epitope is well-distributed on multiple glycoprotein species across all intra-mammalian life stages of the parasite, including immunologically vulnerable cercaria and schistosomula. Future studies should focus on the role of these anti-glycan antibodies *in vivo*, assess their contribution to disease resistance in protected hosts, and determine the suitability of CX/CF-carrying schistosome glycoproteins as vaccine candidates.

## Materials and methods

### Antibodies, microarray and Western blotting materials

Secondary antibodies used for array screening and immunofluorescence were purchased from Invitrogen. Goat anti-mouse IgG-Alexa 488, anti-mouse IgM-Alexa 633 (for DSA); goat anti-rat IgG-Alexa 546 (A11081), goat anti-rat IgM-Alexa 488 (A21212) (for DSA and CFG); anti-mouse IgM-Alexa 488 (A21042), anti-mouse IgG-Alexa 568 (A11031) (for CFG); goat anti-rabbit IgG-Alexa 488 (DSA and immunofluorescence). HRP-conjugated secondary anti-mouse and anti-rabbit antibodies and streptavidin-HRP used for Western blotting and ELISA were purchased from KPL (Gaithersburg, MD). SuperSignal West chemiluminescent substrates were purchased from Thermo Scientific (Rockford, IL). N-hydroxysuccinimide (NHS)-activated slides were purchased from Schott (Elmsford, NY). Rabbit anti-rat IgG-HRP was purchased from Dako (P045001-8). Anti-horseradish peroxidase (HRP) antibody (polyclonal rabbit IgG) was purchased from Sigma-Aldrich (P7899). IgG from rabbit serum (used as a negative control) was purchased from Sigma (I5006).

### Culture and parasite assay materials

Percoll was purchased from GE Healthcare (Piscataway, NJ). Dulbecco’s modified Eagle’s medium (DMEM) was purchased from Cellgro (Manassas, VA). Penicillin-streptomycin was purchased from Gibco (Grand Island, NY). Standard guinea pig complement was purchased from Cedarlane (Burlington, NC). Fetal bovine serum was purchased from Atlanta Biologicals (Lawrenceville, GA). 4’6-Diamidino-2-phenylindole (DAPI) was purchased from Life Technologies (Foster City, CA). Ninety-six-well microplates were purchased from Greiner Bio-One (Frickenhausen, Germany).

### Glycoproteins and glycopeptide preparation

Glycoproteins containing N-glycan core modifications were obtained as follows: Peroxidase from horseradish Type VI and Type I were purchased from Sigma (P8375; P8125). Lectin from *Phaseolus vulgaris* (red kidney bead) phytohemagglutinin (PHA-P) (Sigma), Phospholipase A2 from honeybee venom (*Apis mellifera*) (PLA-2) (Sigma), and Pineapple Bromelain (BRO) was purchased from Sigma.

HRP glycopeptides were prepared *via* pronase digestion of HRP. Pronase was obtained from Calbiochem. 250 mg of HRP was dissolved in 50 mL 5 mM Tris HCl buffer, pH 8.5 with 3 mM CaCl_2_. 50 mg pronase was added to 1 mg/mL with 10 μL toluene. The mixture was incubated around 50°C for 48 h, adding an additional 50 mg pronase at 24 h. The digest was boiled for 10 min and then centrifuged to remove debris. Supernatant was run through two activated 2 g C-18 columns in series to remove undigested protein and glycopeptides were collected in the flow through. Glycopeptides were lyophilized and then desalted and further purified using two 1 g Carbograph columns in series. Fractions eluted from the carbograph using 30%, 50%, and 80% ethanol were pooled and dried, but were found to require further purification in order to profile *via* MALDI mass spectrometry. The glycopeptides were further separated using a Biogel P2 column to enrich for fractions with high hexose to protein ratio. All fractions were quantified for total dry weight, protein content using A280 nm, and hexose content using the phenol sulfuric acid assay. The fractions with highest hexose:protein ratio were pooled. The pool used for further experiments as HRP glycopeptides was 27.8 mg total, 80% hexose by weight and accounted for approximately 40.5% yield of the carbohydrate content of the original protein. MALDI profiling confirmed the presence of several glycopeptides which all contained N-glycopeptides bearing core xylose and core fucose, matching the HRP pronase glycopeptide weights found in Song *et al.*, and which contained at most 2 amino acids (Song et al., 2009). The highest abundance glycopeptide matched the composition Hex3HexNAc2Fuc1Xyl1-NR.

### Animal infections

Sera from *S. mansoni*-infected rats were collected by one of the authors, Charles Shoemaker at Tufts University Cummings School of Veterinary Medicine. Male Fischer rats were infected with 1,000 *S. mansoni* cercariae (high dose) and re-infected at 4 weeks, at which time point it had previously been demonstrated that most adult worms were cleared (Sepulveda et al., 2010). Pooled rat sera were obtained at 4 weeks post-secondary infection from 5 animals.

Mice infected with *S. mansoni* were kindly provided by the Schistosome Research Reagent Resource Center (Rockville, MD) for distribution by BEI Resources, NIAID, NIH. Female Swiss-Webster mice (4–6 weeks old) from Taconic Farms were infected with an average of 30 (low dose) or 200 (high dose) cercariae per mouse, shipped to our facility, and maintained under an approved IACUC protocol at Emory University. Infected mice were monitored for abdominal distention and piloerection and sacrificed if experiencing excessive stress. High dose-infected mice were sacrificed at 7.5 weeks post-infection for collection of adult worms and eggs, and low-dose infected mice were sacrificed at 20 weeks post infection. Sera were pooled from 5 animals in each group at 2 weeks, 4 weeks and 6 weeks; 8 weeks and 20 weeks sera were obtained from low dose infected animals only. All sera were obtained by facial vein puncture except the chronic infection sera, which was obtained by cardiac puncture following euthanasia. At the conclusion of our experiments, all infected mice were euthanized by intraperitoneal overdose with 300 μl of 65 mg/ml sodium pentobarbital with 200 U/ml heparin sodium salt. All experiments involving mice were approved by the Emory University IACUC.

Rhesus monkey sera were harvested from *M. mulatta* infected percutaneously with 500 cercariae, and sera were collected during the course of infection at various time points from 4 to 21 weeks post infection, or 11 weeks after a second infection, from an IACUC-approved study conducted at the Division of Parasitic Diseases and Malaria at the National Centers for Disease Control and Prevention (CDC). Infections were monitored by the examination of eggs in stool samples.

### Array binding assays

The Defined Schistosome-type Array (DSA) was prepared and validated as previously described (Heimburg-Molinaro et al., 2011; Luyai et al., 2014; Prasanphanich et al., 2014). The printing of glycan arrays was performed using a Piezorray printer (PerkinElmer, Waltham, MA), and the analysis of glycan arrays was accomplished by scanning with a ProScanArray Scanner (PerkinElmer) equipped with 4 lasers. Binding assays were performed as per Heimburg-Molinaro *et al.* for experiments with rabbit anti-HRP (Heimburg-Molinaro et al., 2011). For binding assays with animal sera, rat and mouse serum was diluted 1:50 in TSM binding buffer (20 mM Tris-HCl pH 7.4, 150 mM sodium chloride, 2 mM calcium chloride, 2 mM magnesium chloride, 0.05% tween-20, 1% BSA) for a 1 h incubation with rocking, and wells were washed 3 times with 200 μl TSM wash buffer (20 mM Tris-HCl pH 7.4, 150 mM sodium chloride, 2 mM calcium chloride, 2 mM magnesium chloride, 0.05% tween-20), then 3 times with 200 μl TSM buffer (20 mM Tris-HCl pH 7.4, 150 mM sodium chloride, 2 mM calcium chloride, 2 mM magnesium chloride) with 5 min of shaking for each wash, after the primary and secondary incubations. Secondary antibodies, goat anti-mouse IgG-Alexa 488, and anti-mouse IgM-Alexa 633, goat anti-rat IgG-Alexa 546 and anti-rat IgM-Alexa 488 were used at 5 μg/mL for 1 h.

For HRP blocking experiments on the DSA, 100 μg of HRP that had previously been boiled was added to the naïve or infected sera diluted in array binding buffer and rocked for 10 min at room temperature before adding to the array chambers.

Binding assays on the CFG array version 5.1 were conducted as described (Heimburg-Molinaro et al., 2011) with the following modifications: mouse and rat sera were used at 1:50 in 70 μl of binding buffer for the primary incubation. After each incubation, the slide was dipped 3 times and rocked for 3 5 min incubations in wash buffer with tween, and the same procedure was then repeated in wash buffer without tween. Naïve mouse serum and naïve rat serum were run first and, once scanned and verified to be negative, slides were re-used for infection anti-sera.

Slides were scanned using the ScanArray Express software on a PerkinElmer Proscanner XL4000. ScanArray Express was used to align spots, remove background and quantify fluorescence. An excel macro file was then used to average 4 replicate spots for each glycan ID # and determine SEM, SD and %CV. Glycopattern was used to visualize top binders and compare heat map results among samples on the CFG glycan microarray (Agravat et al., 2014).

### Isolation of *Schistosoma mansoni* life stages and preparation of parasite lysates

*Schistosoma mansoni*-infected *B. glabrata* snails were kindly provided by the Schistosome Research Reagent Resource Center for distribution by BEI Resources, NIAID, NIH: *S. mansoni*, strain NMRI NR-21962. All work with *B. glabrata* and *S. mansoni* was approved by the Emory University Office of Occupational Health and Safety and conducted in biosafety level II animal surgery facilities and laboratories in compliance with university-approved biosafety and IACUC protocols.

Snail maintenance and collection of cercariae was as per unit 19.1 of *Current Protocols in Immunology*, “Schistosomiasis” (Lewis, 1998) with modifications described previously (Prasanphanich et al., 2014)*.* Schistosomula were prepared by vortexing cercariae for 45 s, icing 3 min, and vortexing another 45 s to remove tails. Schistosomula were isolated by spinning at 1,500 rpm for 10 min through a 60% Percoll solution in PBS and washing the schistosomula pellet twice in cold DMEM with Penn/Strep, with 1,000 rpm spins. The schistosomula were cultured for 3 h—3 days at 37°C with 5% carbon dioxide in DMEM with 10% FBS, penicillin/streptomycin at a density of approximately 1,000 organisms/mL in tissue-culture dishes. Preparation of adult worms and eggs, and soluble egg antigen was as per Lewis *et al.* (Lewis, 1998) and with modifications described previously (Prasanphanich et al., 2014)*.*

Cercarial secretions and section depleted pellet were made based on the protocol of Curwen *et al.* (Curwen et al., 2006). Vortex-transformed cercaria were incubated in DMEM with penicillin/streptomycin for 3 h and centrifuging at 130xg for 8 min. An equivalent volume of water harboring uninfected snails was cultured and prepared in the same way to make the cercarial secretions control. The secretion-depleted cercarial pellet was made by lysing the pellet that remained after spinning the above culture. All work with *B. glabrata* and *S. mansoni* was approved by the Emory University Office of Occupational Health and Safety, and conducted in BSL-II animal surgery facilities and laboratories.

For preparation of parasite lysates, chilled pelleted cercariae, schistosomula or cercarial secretions were transferred to 1.5 mL Eppendorf tubes in at most 50 µl of Triton-X parasite lysis buffer (50 mM Tris buffer pH 8.0, 2.5% 2-mercaptoethanol, 1% Triton-X100, 1 mM EDTA and 1 tablet of Complete Mini protease inhibitor per 10 mL of lysis buffer) per 10,000 cercariae or 25 µl of parasite lysis buffer per 10,000 schistosomula. This was vortexed, boiled for 15 min (vortexing once during the boiling incubation), and centrifuged at 20,000xg for 2 min. The supernatant was removed to a clean tube, and a small amount of lysis buffer was added to the pellet for another 10 min boiling incubation, after which the spin was repeated and the supernatants were pooled. RIPA extracts were made in a similar fashion, except washed pellets were resuspended in cold RIPA buffer (25 mM Tris HCl pH 7.6, 150 mM NaCl, 1% NP-40, 1% sodium deoxycholate, 0.1% SDS), vortexed briefly, sonicated 4 times in 5 s pulses, incubated shaking for 15 min on ice, and then spun at 14,000xg for 15 min. The supernatant was removed and Complete Mini protease inhibitor was added.

Adult worm lysate was made by bringing a freshly-thawed adult worm pellet of approximately 0.5 mL up in 5 mL of PBS, spinning at 500xg for 10 min at 4°C, and adding 3 mL of lysis buffer to the pellet. The worm lysate was made as described above except 1% SDS and 1 mM phenylmethylsulfonylfluoride (PMSF) were used instead of Triton-X100 and the Complete Mini tablet, respectively, and the SDS was salted out using 100 mM KCl so the lysates could be concentrated in Amicon spin filter tubes (3,000 Da MWCO). Triton-X100 was then added back to 1% to the concentrated adult worm lysates. Soluble egg antigen was prepared as per unit 19.1, “Schistosomiasis,” of *Current Protocols in Immunology* (Lewis, 1998). Parasite lysates were quantified by the Pierce 660 nm and BCA protein assays and stored in aliquots at −80°C.

Periodate treatment of parasite lysates in solution was accomplished by dialyzing cercarial lysate into water overnight to remove buffer and reducing agents and then adjusting the buffer to 0.1 M sodium acetate, 0.15 M NaCl, pH 5.5. Sodium m-periodate was added to 20 mM and the mixture was rotated in the dark at 4°C overnight. The reaction was quenched with a molar excess of sodium borohydride. The reactions were again dialyzed to water and concentrated.

### Immunofluorescence of whole parasites

Using cercaria or schistosomula isolated as described above and cultured schistosomula, parasites were washed 3 times in ice cold PBS, spinning at 100xg with brakes on the lowest possible setting. They were resuspended in a small amount of PBS and then brought up in 10 mL of 10% neutral buffered formalin and allowed to rotate slowly in the dark at 4°C for 16–24 h. These were washed again 3 times in cold PBS as described above and stored at 4°C until use. For immunostaining, approximately 500 parasites were used for each sample and all incubations were 1 h, slowly rotating at 4°C in 200 µl of 3% BSA in PBS for blocking or antibody diluted in 3% BSA in PBS. Washes were in 200 µl cold PBS after spinning 3 min at 100xg. The parasites were blocked for 1 h and then re-suspended in 100 μg/mL rabbit anti-HRP (Sigma). After primary incubation they were washed 4 times and re-suspended in goat-anti-rabbit IgG-Alexa 488 (Invitrogen) at 1:500. After secondary incubation, the parasites were washed 3 times and transferred in the remaining 10–20 µl to a glass slide and 10 µl of mounting medium (Southern Biotech) was added and the coverslip was applied. Where noted, the parasites were imaged directly in the 96-well plate. Parasites were imaged on an Olympus microscope.

### *In vitro* schistosomula killing assays

Schistosomula were isolated as described above and cultured in 96-well plates with 50–100 parasites in 35 µl of warmed DMEM with penicillin/streptomycin in each well at 37°C. At 3 h, the specified antibodies or animal sera were then added, diluted in 17.8 µl DMEM (antibodies at 0.5–2.0 mg/mL). At 3.5 h, 15 µl of freshly-thawed, active or heat inactivated (1 h at 55°C) guinea pig complement was added (final concentration 1:5) to each well along with 7.5 µl fetal bovine serum (final concentration 10%) and penicillin/streptomycin to final concentration of 100 U/ml. Each antibody or serum killing condition was assayed in duplicate or triplicate wells. The total number of live schistosomula were counted at 0 h. At approximately 22 h, 1 µl of 100 μg/ml DAPI stain was added to each well. DAPI positive schistosomula were counted as dead at 24, 48 and 72 h.

If a glycoprotein or glycopeptide was included to block antibody activity, it was pre-incubated with the antibody or serum preparation at 0.67–2.67 mg/mL for 1 h before addition to schistosomula. Glycoproteins were pre-boiled for 20 min to destroy any enzymatic activity. Killing assays with naïve or *S. mansoni*-infected animal sera were performed exactly as described, except mouse sera at 1:25–1:50, rhesus sera at 1:25–1:75, or rat sera at 1:50–1:75 were diluted in 17.8 µl DMEM per well.

### SDS-PAGE and Western blots

For SDS-PAGE, 5–15 µg per well of parasite or 2–3 µg/well of glycoproteins (BSA and HRP) lysates (lanes within the same figure were equally loaded unless specified in the labels) were boiled in 1x NuPAGE SDS sample buffer +2.5% *β*-mercaptoethanol for 10 min and then run in Mini-PROTEAN-TGX gels at 200 V for 30 min, with 7 µl of protein standards. Protein was transferred to a nitrocellulose membrane using the 10-min high molecular weight program, 7-min mixed molecular weight program or 30-min standard program in the Trans-Blot Turbo semi-dry transfer system. All subsequent incubations were shaking at ambient temperature. Membranes were stained with 0.1% Ponceau S in 5% acetic acid to check for equal loading and transfer, and destained with 1X TBS-t wash buffer (20 mM Tris, 300 mM NaCl, 0.1% Tween-20). At this point the membrane was sometimes stored overnight at 4°C in TBS.

To silver stain, SDS-PAGE gels were placed in destaining solution (50% ethanol, 10% acetic acid) for 1 h and then washed in 50% ethanol 3 times, for 20 min each. All incubations were with shaking. The gel was then placed in pre-treatment solution (0.02% sodium thiosulfate) for 1min and washed 3 times, 20 s each in distilled water. It was then placed in impregnating solution (0.2% silver nitrate with 0.03% formaldehyde) for 20 min. The gel was then washed 3 times in distilled water for 20 s each and placed in developer solution (6% sodium carbonate, 0.02% formaldehyde, 0.0004% sodium thiosulfate) until bands appeared and destained.

For staining with serum, membranes were blocked for 2 h in 2% (w/v) de-fatted dried milk in 0.5X TBS-t (10 mM Tris, 150 mM NaCl, 0.05% tween), followed by one 10 min wash in 1X TBS-t. Sera were diluted 1:1000 in milk diluent (0.5% milk in 0.5X TBS-t) and incubated with the membranes for 1 h. The membranes were then washed 3 times quickly and 3 times for 5 min each in 1X TBS-t. Secondary detection antibodies (HRP-conjugated goat anti-mouse-IgG or–IgM) were added for 1 h at 1:5000 (mouse) or 1:3000 (rat) in milk diluent. The same wash procedure was repeated, and then Super Signal West Pico Chemiluminescent Substrate was added for 30 s. The membranes were dabbed dry and exposed to film for 1 s-3 min (exposure times are indicated when this varied for panes shown in the same figure).

When blotting was performed on glycoproteins including BSA, HRP, BRO, PLA2 and PHA, a similar protocol was followed except the primary anti-HRP antibody was diluted to 1 μg/mL, the secondary anti-rabbit antibody was used at 1:10000; and rat serum was diluted to 1:500 with secondary detection at 1:1000. Three quick washes were followed by 3 × 5 min washes were performed after each antibody incubation.

When periodate-treated lysates were stained with sera, anti-rat and anti-mouse secondaries were both used at 1:3000.

### ELISA

Horseradish peroxidase type VI (HRP) (Sigma, P8375) was permanently denatured to destroy endogenous peroxidase activity before use in ELISA, or boiled before SDS-PAGE/Western blots. HRP was dissolved in 0.2 M Tris-HCl with 8 M guanidine HCl and 60 mM dithiothreitol and rolled for 1 h at room temperature. Iodoacetamide was added to 100 mM and incubated for another 30 min, followed by dialysis of the HRP into water. It was verified that the denatured HRP gave no background signal from endogenous enzyme activity at the coating concentrations used.

HRP was coated on ELISA plates at 0.5 μg/mL in 0.05 M carbonate/bicarbonate buffer pH 9.6, 50 μl per well, in a clear, flat-bottom, non-tissue culture treated 96-well plate (Greiner, 655101) overnight at 4°C. Excess solution was discarded and wells were washed 3 times in PBS-t (0.05% tween-20). Sodium acetate 0.1 M, pH 5.5 was added, 100 μl per well, for 30 min, and then 20 mM sodium-m-periodate in sodium acetate buffer, or buffer alone for mock-treatment, 100 μl per well, was added for an overnight incubation at 4°C in the dark. The periodate solution was then discarded and all wells were quenched with 150 μl of 0.1 M sodium borohydride solution in PBS-t for 30 min at room temperature. Wells were then washed 3 times with PBS-t using a squirt bottle and the ELISA was performed.

Plates were blocked with 200 μl per well of 3% BSA in PBS-t. All incubations were for 1h at room temperature with rocking. Primary and secondary antibodies were made up in PBS-t with 1% BSA (1–10 μg/mL for rabbit anti-HRP; 1:1000 for HRP-conjugated goat anti-rabbit IgG and 50 μl was added to each well. For detection, 1 tablet of O-phenylenediamine dihydrochloride was dissolved to 0.5 mg/mL in stable peroxide buffer (Thermo) and 50 μl was added to each well, and incubated, standing, in the dark for 10 min. To quench the reaction, 25 μl of sulfuric acid was added. The plate was read at 490 nm on a Victor plate reader.

### Immunoprecipitation of glycoproteins with rabbit anti-HRP

Rabbit anti-HRP, and naïve rabbit IgG (as a negative control) were conjugated to Ultralink Biosupport beads (Thermo Cat #53110) at 4°C, as per manufacturer protocol, except as noted. Briefly, 2.5 mg of each antibody was coupled to 10 mg of beads in 0.1 M MOPS/0.6 M sodium citrate buffer, pH 7.5, in total reaction volume of 0.5 mL for 2.5 h. The supernatant was removed and the reaction was quenched by adding 1 mL 3.0 M ethanolamine pH 9.0. Beads were washed and subsequently stored in phosphate buffered saline.

Immunoprecipitation of parasite lysates was performed both with and without initial blocking of the beads (in 10% normal human serum/RIPA parasite lysate buffer for 30 min at room temperature), however results were the same, so only the unblocked condition is shown. Beads were washed 3 times in RIPA. Roughly 200 µg of 3-h schistosomula lysate (previously frozen at −80°C) was combined with 100 µl of the above Ultralink beads in a total reaction volume of 1.2 mL and incubated for 2.5 h at 4°C, rolling. The supernatant (unbound) was removed, and beads were washed 3x in RIPA buffer. To elute the bound species, 50 µl of LDS-BME buffer was added and beads were boiled. Small aliquots of each bound and unbound fraction were used for SDS-PAGE, silver stain and confirmatory Western blots as detailed above, and the remainder of the bound fractions were used for proteomics analysis.

## Data Availability

The raw data supporting the conclusion of this article will be made available by the authors, without undue reservation.
