# Supplementary material for: Anti-schistosomal immunity to core xylose/fucose in N-glycans
Source: Front Mol Biosci. 2023 Apr 4;10:1142620. doi: 10.3389/fmolb.2023.1142620 (PMC10110957; doi:10.3389/fmolb.2023.1142620)
Supplement: Supplementary file 1 [file DataSheet1.DOCX]

Prasanphanich et al.

**Supplementary Information**

**Supplementary Figure Legends**

**Fig. S1. Glycan structures of the Defined Schistosome-type Array (DSA).**

**Fig. S2. Secondary-infected rat IgG has broad anti-glycan targeting, including antibodies to Lewis X and poly-N-acetyllactosamine.** Rat serum from 4 weeks post *S. mansoni* re-infection (**a**) and mouse serum from 4-20 weeks (**b-e**) post infection binding to the Consortium for Functional Glycomics (CFG) v5.1 array. Anti-mouse IgG-Alexa 568 (**b-e**) and anti-rat IgG-Alexa 546 (**a**) used for detection of IgG. Each bar represents an average of binding intensity (relative fluorescence units, RFU) to hexa-replicate spots with the high and low value removed for each glycan ID number (listed on X-axis and above selected bars) +/- 1 standard deviation. Top binding structures depicted, see monosaccharide key in **Fig. S1**. Full listing of glycans can be found at the CFG and NCFG website*.*

**Fig. S3. Secondary-infected rat IgM targets Lewis X and LDNF.** Rat serum from 4 weeks post *S. mansoni* re-infection (**a**) and mouse serum from 4-20 weeks (**b-e**) post infection binding to the CFG v5.1 array. Anti-mouse IgM-Alexa 488 (**b-e**) and anti-rat IgM-Alexa 488 (**a**) used for detection of IgM. Each bar represents an average of binding intensity (relative fluorescence units, RFU) to hexa-replicate spots with the high and low value removed for each glycan ID number (listed on the X-axis and above selected bars) +/- 1 standard deviation. Top binding structures depicted, see monosaccharide key in **Fig. S1**. Full listing of glycans can be found at the CFG and NCFG website*.*

**Fig. S4. Rat and mouse antisera targeting of *S. mansoni* glycoproteins. a**) *S. mansoni* life stage lysates, normalized to total protein content, run on SDS-PAGE and Western blots stained with pooled twice-infected rat serum (center panel) and 8-week infected mouse serum (right panel) at 1:1000. Soluble egg antigen, E; adult worms, A; cercaria, C. Naïve rat sera shown (left panel) as a negative control. **b**) Cercarial lysate treated with (C+p) or without (C) periodate and run on SDS-PAGE and silver stained to verify proteins were not degraded (left panel). Treated cercarial lysates blotted with biotinylated *Aleuria aurantia* Lectin (AAL) to verify loss of glycan reactivity (middle panel), and stained with infected mouse and rat serum (middle and right panels). **c)** Rat and mouse serum binding to soluble egg antigen in ELISA, with or without in-plate periodate treatment (tx).

**Fig. S5. Rabbit anti-HRP and infected rat serum bind similar glycan epitopes of HRP and other glycoproteins. a**) Polyclonal rabbit anti-HRP IgG analyzed for binding to HRP-coated ELISA plates, treated with (light bar) or without (dark bar) periodate. **b**) rabbit anti-HRP and infected rat serum staining of plant glycoproteins containing versions of CX and CF epitopes show a similar pattern. PHA: phytohemagglutinin; PLA-2: Phospholipase A2 from honeybee venom; BRO: Pineapple Bromelain; BSA: Bovine serum albumin; HRP: Horseradish peroxidase.

**Fig. S6. Immunoprecipitation of CX/CF-carrying glycoproteins from schistosomula lysate.** Beads covalently linked to either rabbit anti-HRP (α-HRP) or normal rabbit IgG (ctrl) used to immune-precipitate CX/CF-containing glycoproteins from schistosomula lysate. Fractions that either bound (B) or remained in supernatant (U, unbound) run on SDS-PAGE gels and blotted with anti-HRP. Red boxes highlight schistosomula glycoprotein species which bound to α-HRP-linked beads, but not to control beads, indicating enrichment for particular species.

**Fig. S7. Correlations between *in vitro* schistosomula killing activity of rhesus monkey sera and glycan reactivity.** Sera from individual infected rhesus monkeys each tested in duplicate in schistosomula killing assays as described for **Fig. 5** and 48 hour % schistosomula killing plotted on X-axis. The RFU of IgG or IgM binding from each of these sera at 1:50, to tetra-replicate spots of each glycan ID on the DSA (see **Fig.** **S1** for glycan ID#s and structures) is plotted in log scale on Y-axis. Three samples of X-Y scatter plots shown to demonstrate a moderate, significant correlation (left), a weak, non-significant correlation (middle) and zero correlation (right). Pearson correlation coefficient (r) shown for each glycan, with level of significance by P value, and color coded by strength of the correlation in the heat map.

**Fig. S1.**


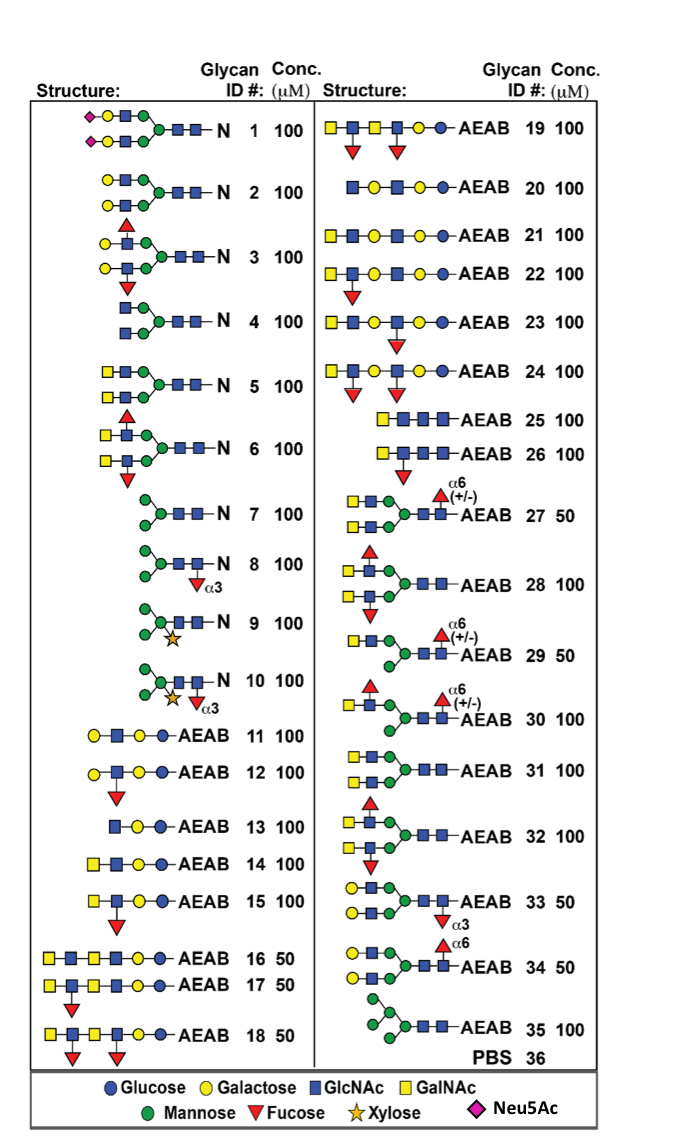


**Fig. S2.**

**Fig. S3.**

**Fig. S4.**

**Fig. S5.**

**Fig. S6.**

**Fig. S7.**
